# Prognostic value of microRNA-21 in pancreatic ductal adenocarcinoma: a meta-analysis

**DOI:** 10.1186/s12957-016-0842-4

**Published:** 2016-03-11

**Authors:** Geng-yuan Hu, Feng Tao, Wei Wang, Ke-wei Ji

**Affiliations:** Department of Gastrointestinal Surgery, Shaoxing People’s Hospital, Shaoxing Hospital of Zhejiang University, No. 568, Zhongxing North Road, Shaoxing, 312000 China

**Keywords:** MicroRNA-21, Prognosis, Pancreatic ductal adenocarcinoma, Meta-analysis

## Abstract

**Background:**

Recently, microRNA-21 (miR-21) has been reported to be associated with prognosis of pancreatic ductal adenocarcinoma (PDAC). The present studies aimed to evaluate the prognostic value of miR-21 for PDAC with meta-analysis.

**Methods:**

A systematic search in the PubMed and other databases was conducted to identify eligible studies. The pooled hazard ratios (HRs) with 95 % confidence interval (CI) were calculated. The meta-analysis was conducted using the STATA 12.0 software.

**Results:**

A total of 12 articles (13 studies) which included 963 cases were selected for the meta-analysis. Elevated miR-21 expression was significantly predictive of poor overall survival (HR = 2.05, 95 % CI 1.71–2.46, *P* < 0.001). In the subgroup analyses, similar results were observed in Asian (HR = 2.09, 95 % CI 1.62–2.71, *P* < 0.001) and Caucasian (HR = 2.36, 95 % CI 1.53–3.65, *P* < 0.001); in tissue sample (HR = 2.14, 95 % CI 1.73–2.65, *P* < 0.001) and serum sample (HR = 1.84, 95 % CI 1.30–2.60, *P* = 0.001); with quantitative real-time polymerase chain reaction assay method (HR = 2.31, 95 % CI 1.86–2.86, *P* < 0.001); and in patients receiving adjuvant chemotherapy (HR = 2.37, 95 % CI 1.88–3.00, *P* < 0.001). The association between miR-21 expression level and lymph node metastasis was statistically significant (OR = 1.45, 95 % CI 1.02–2.06, *P* = 0.038). However, no significant relationship between miR-21 expression level and sex or vascular invasion or neural infiltration was observed (*P* > 0.05).

**Conclusions:**

Our meta-analysis indicated that elevated miR-21 expression level can predict poor prognosis in patients with PDAC.

## Background

Pancreatic ductal adenocarcinoma (PDAC) is one of the most aggressive malignancies and the fourth cause of cancer-related death [1]. Despite the advancement in medical and surgical managements, the outcome of PDAC remains disappointing. Only 15 % of patients can undergo radically surgical resection [2] and approximately 6 % of patients can survive for 5 years following diagnosis [1]. The poor prognosis of PDAC primarilyattributes to the facts including late clinical manifestation, aggressive local invasion, high metastatic potential, and chemotherapy or radiotherapy resistance. These challenges motivate researchers to seek better approaches for PDAC, including finding out new biomarkers which can identify different phenotypes with differences in clinical feature and prognosis.

MicroRNAs are a class of small non-coding RNA, approximately 20 nucleotides in length [3]. By base pairing with the 3′-untranslated region (UTR) of target messenger RNAs, microRNAs act as endogenous regulators of protein-coding genes at transcriptional or post-transcriptional level [3, 4]. Thus, microRNAs (miRNAs) are involved in various biological processes, such as differentiation, proliferation, cell cycle regulation, and metabolism [5, 6]. More importantly, mounting evidences suggest that microRNAs are involved in cancer development and progression including activation of oncogenes and inactivation of tumor suppressor genes [7, 8].

MiR-21 is the most frequently observed cancer-related microRNA. Previous studies have observed that miR-21 dysregulates in many cancers and acts as a key factor mediating the growth, development, and progression of tumors [9–11], which is expected to be a novel predictor and target. However, evidence for the prognostic role of miR-21 expression in PDAC was still lacking. Thus, we conduct this meta-analysis to evaluate the prognostic value of miR-21 for PDAC.

## Methods

### Search strategy

A systematic search in the PubMed, ISI Web of Science, and EMBASE databases was performed to identify studies published before February 2016. The detailed keywords for the PubMed searches were as follows:

1. miR-21.
2. miRNA-21.
3. microRNA-21.
4. pancreatic cancer.
5. pancreatic tumor.
6. pancreatic carcinoma.
7. prognos*.
8. survival.
9. outcomes.
10. #1 OR #2 OR #3
11. #4 OR #5 OR #6
12. #7 OR #8 OR #9
13. #10 AND #11 AND #12

**Fig. 1:**
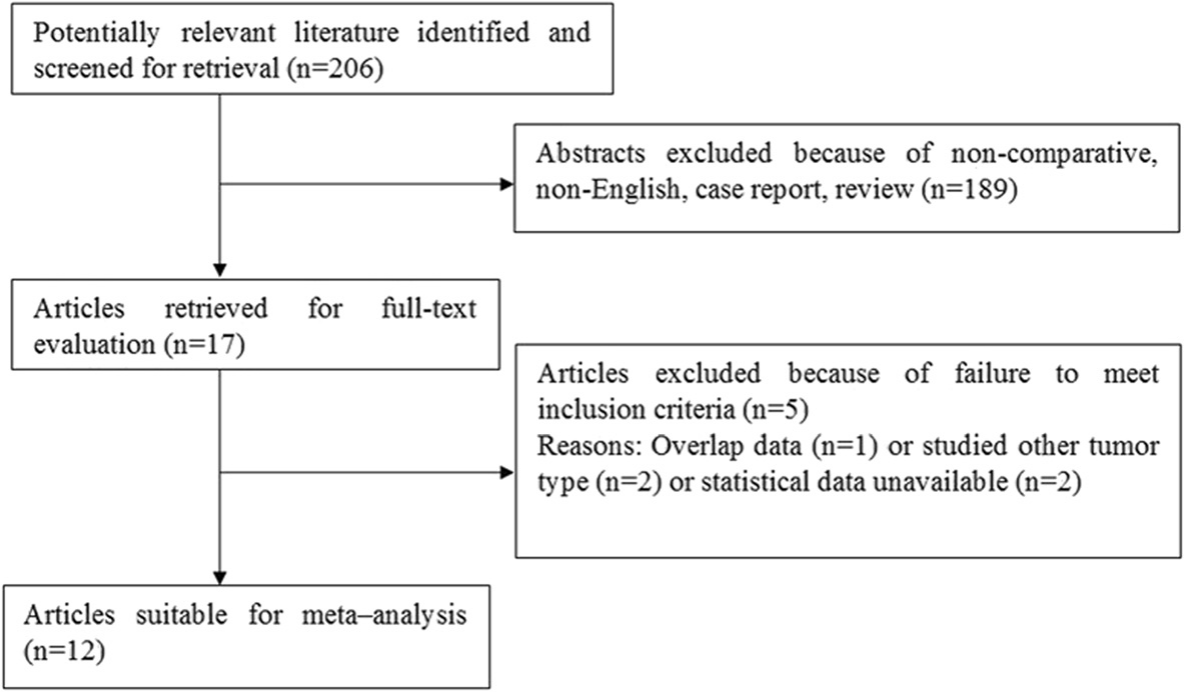
The PRISMA flowchart of literature review

All of the studies which fulfilled the following criteria were included in the meta-analysis: (1) studied PADC based on histopathological confirmation; (2) expression of miR-21 was measured; (3) the association between expression of miR-21 level and survival outcome was studied. Studies were excluded based on the following criteria: (a) reviews, letters or laboratory studies, unpublished studies with only the abstracts presented at national and international meetings; (b) studies had overlapping or duplicate data; (c) absence of necessary information to calculate HR. All references of retrieved articles were manually selected to identify all the potential studies. The language of the articles was limited to English. A flow diagram of the study selection process is presented in Fig. 1.

### Data extraction

Two reviewers (FT and KWJ) independently extracted the data following the guidelines of a critical review checklist of the Dutch Cochrane Centre proposed by Meta-analysis of Observational Studies in Epidemiology (MOOSE) [12] and decided upon the controversial issues through discussion. For disagreements, a consensus was reached by a third investigator (WW). The following information was extracted from each study: author, published year, country or area, ethnicity, sample size, sample type, TNM stage, detection method, cutoff value, follow-ups, adjuvant therapy, HRs with 95 % confidence intervals (CIs), and *P* value of miR-21 for overall survival (OS). If not available, data were extracted using the method described by Tierney et al. [13].

### Statistical analysis

Pooled hazard ratio (HR) of miR-21 expression for OS was calculated. The heterogeneity was checked using *I*^2^ statistic described by Higgins [14]. A *P* < 0.05 or *I*^2^ > 50 % for *Q* test suggested significant heterogeneity among studies. If high heterogeneity exists among studies, pooled effect was calculated using the random-effects model (DerSimonian–Laird method) [15], Otherwise, the fixed-effects model (Mantel–Haenszel method) was used [16]. Publication bias was assessed using the funnel plot. Subgroup analysis of PADC patients with elevated miR-21 expression were examined with respect to gender (male vs. female), lymph node metastasis (positive vs. negative), vascular invasion (positive vs. negative), and neural infiltration (positive vs. negative). Data analyses were performed using STATA software version 12.0 (STATA Corporation, College Station, TX, USA). *P* < 0.05 was considered statistically significant.

## Results

### Summary of included study characteristics

A total of 206 published articles were initially retrieved in the databases of PubMed, EMBASE, and Web of Science. After abstract review, 189 articles were removed which left 17 articles for full-text evaluation. Finally, 12 articles (13 studies) remained which excluded 5 articles following full-text evaluation [17–28], Fig. 1. The main characteristics and results of the eligible studies are summarized in Table 1. These studies investigated a total of 963 cases from USA, China, Italy, UK, Korea, Japan, Greece, and Germany. MiR-21 expression was detected by either quantitative real-time polymerase chain reaction assay (qRT-PCR) or in situ hybridization (ISH) with 6 in FFPE, 2 in cancerous tissue, 2 in frozen tissue, and 3 in serum. The cutoff values of miR-21 varied in each study. Median and mean values were extracted from 9 studies; 2-fold values or score > 2 or score > 1 were considered in the remaining 4 studies and Liu et al. did not report the cutoff value [22]. The technical details in the detection of miR-21 expression are summarized in Table 2.

**Table 1:** The characteristics of the pooled studies

Study	Year	Country	Ethnicity	Sample size	Stage	Results	Survival analysis	HR (95 % CI) for prognostic outcomes	Adjuvant chemotherapy
Dillhoff	2008	USA	Caucasian	80	NR	OS	KM	OS, 4.08 (1.98, 8.38), *P* = 0.037	NR
Giovannetti a	2010	Italy	Caucasian	28	I–III	OS, DFS	U	OS, 3.1 (1.4–7.3), *P* = 0.008	Y
								DFS, 4.4 (1.8–10.7), *P* = 0.001	
Giovannetti b	2010	Italy	Caucasian	31	IV	OS, PFS	U	OS, 3.1 (1.4–7.1), *P* = 0.01	Y
								PFS, 2.4 (1.1–5.3), *P* = 0.03	
Hwang	2010	Korea	Asian	82	II–IV	OS, DFS	M	OS, 2.26 (1.34, 3.80), *P* = 0.002	Y
								DFS, 2.793 (1.466–5.319), *P* = 0.002	
Jamieson	2012	UK	Caucasian	48	II–IV	OS	M	OS, 3.22 (1.21–8.58), *P* = 0.019	Y
Liu	2012	China	Asian	38	I–IV	OS	KM	OS, 2.89 (1.22–6.81), *P* = 0.02	NR
Nagao	2012	Japan	Asian	65	I–IV	OS	U	OS, 2.32 (1.19–4.52), *P* = 0.045	NR
Wang	2013	China	Asian	177	III–IV	OS	M	OS, 1.71 (1.15–2.54), *P* = 0.008	Y
Kadera	2013	USA	Caucasian	145	I–IV	OS	U	OS, 1.1 (0.7–1.6), *P* = 0.7	NR
Papaconstantinou	2013	Greece	Caucasian	88	I–IV	OS	M	OS, 1.72 (1.25–12.3), *P* = 0.019	NR
Ma	2013	China	Asian	78	I–IV	OS	M	OS, 2.60 (1.15–5.87), *P* = 0.021	NR
Dhayat	2015	Germany	Caucasian	91	II	OS, RFS	M	OS, 3.06 (1.37–6.85), *P* = 0.0064	Y
								RFS, 2.25 (1.06–4.77), *P* = 0.0338	
Khan	2015	UK	Caucasian	12	IV	OS, PFS	KM	OS, 1.46 (0.41–5.21), *P* = 0.564	Y
								PFS, 4.7 (1.1–19.7), *P* = 0.032	

*OS* overall survival, *DFS* disease-free survival, *RFS* relapse-free survival, *PFS* progression-free survival, *KM* Kaplan–Meier analysis, *U* univariate analysis, *M* multivariate analysis, *Y* yes, *NR* not reported

**Table 2:** The technical details in detection of miR-21 expression

Study	Sample size	Sample	Sample technique	Method	Endogenous control	Cutoff
Dillhoff	80	FFPE	MDS	ISH	RNU6	Score > 1
Giovannetti a	28	Tissue	MDS	RT-PCR	RNU43	Median
Giovannetti b	31	Tissue	MDS	RT-PCR	RNU44	Median
Hwang	82	FFPE	MDS	RT-PCR	RNU66 or RNU43	Median
Jamieson	48	FT	NR	RT-PCR	RNU6	Median
Liu	38	Serum		RT-PCR	NR	NR
Nagao	65	FFPE	NR	RT-PCR	RNU6	Mean
Wang	177	Serum		RT-PCR	RNU7	Median
Kadera	145	FFPE	NR	ISH	RNU6	Median
Papaconstantinou	88	FFPE	NR	RT-PCR	RNU6	Mean
Ma	78	FT	NR	RT-PCR	RNU6	≥2-fold change
Dhayat	91	FFPE	NR	RT-PCR	NR	Mean
Khan	12	Serum		ISH	NR	Median

*FFPE* formalin-fixed paraffin-embedded, *FT* frozen tissue, *MD* microdissected sample, *ISH* in situ hybridization, *qRT-PCR* quantitative real-time polymerase chain reaction assay, *NR* not reported

### Correlation between miR-21 expression and survival outcome

**Fig. 2:**
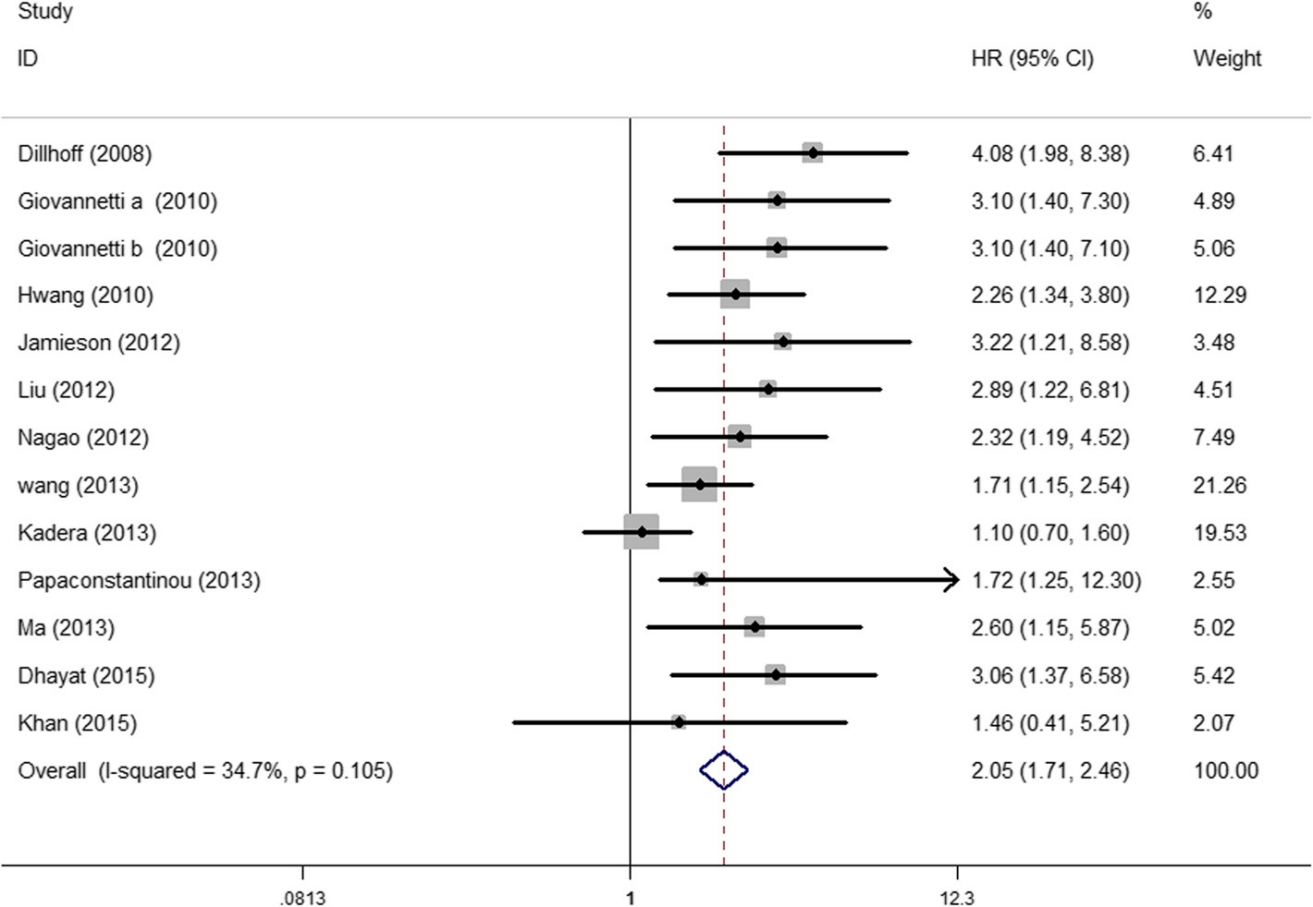
The relationship between elevated miR-21 level and OS

**Fig. 3:**
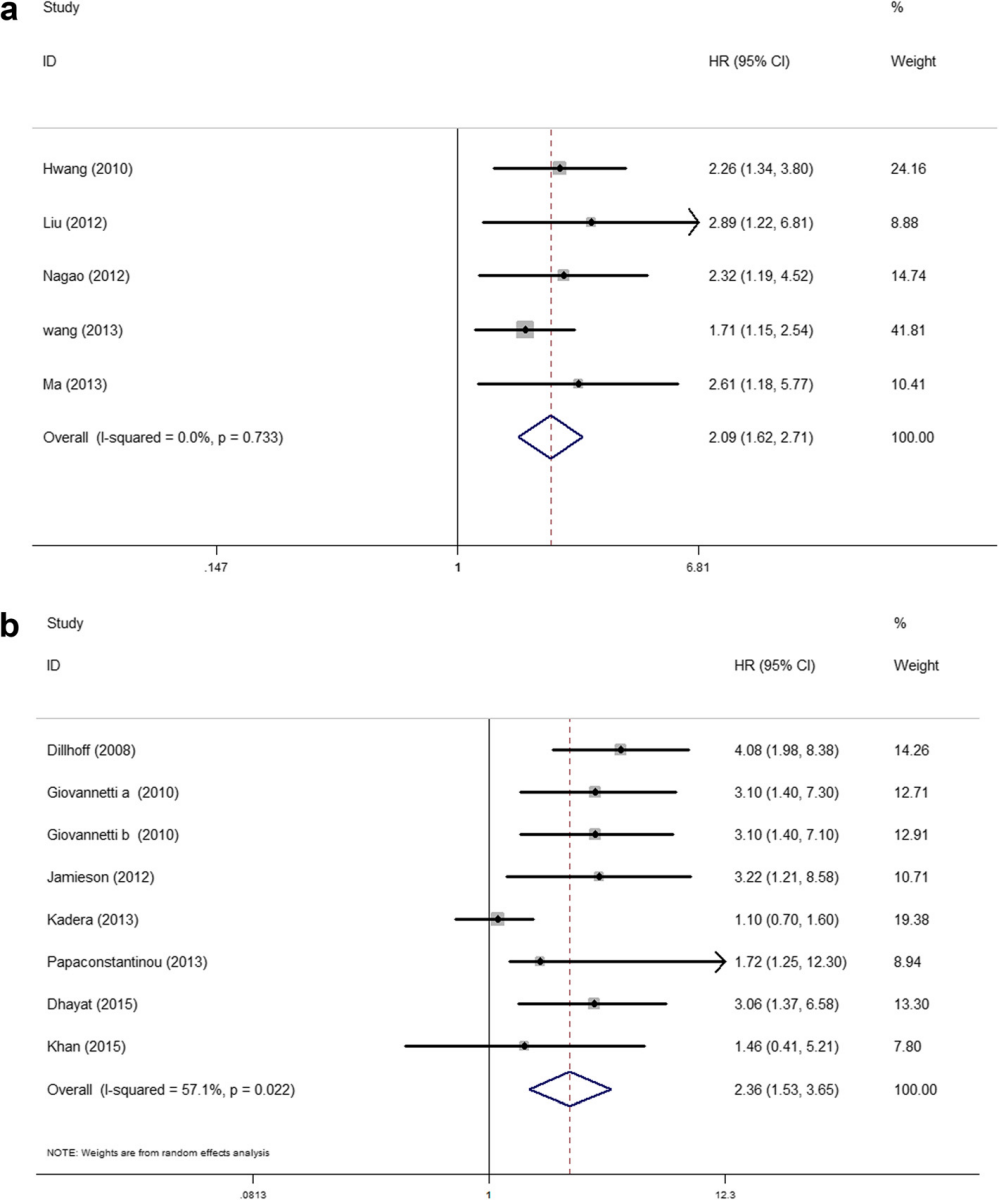
Subgroup analyses of relationship between elevated miR-21 level and OS by ethnicity. **a** Asian. **b** Caucasian

**Fig. 4:**
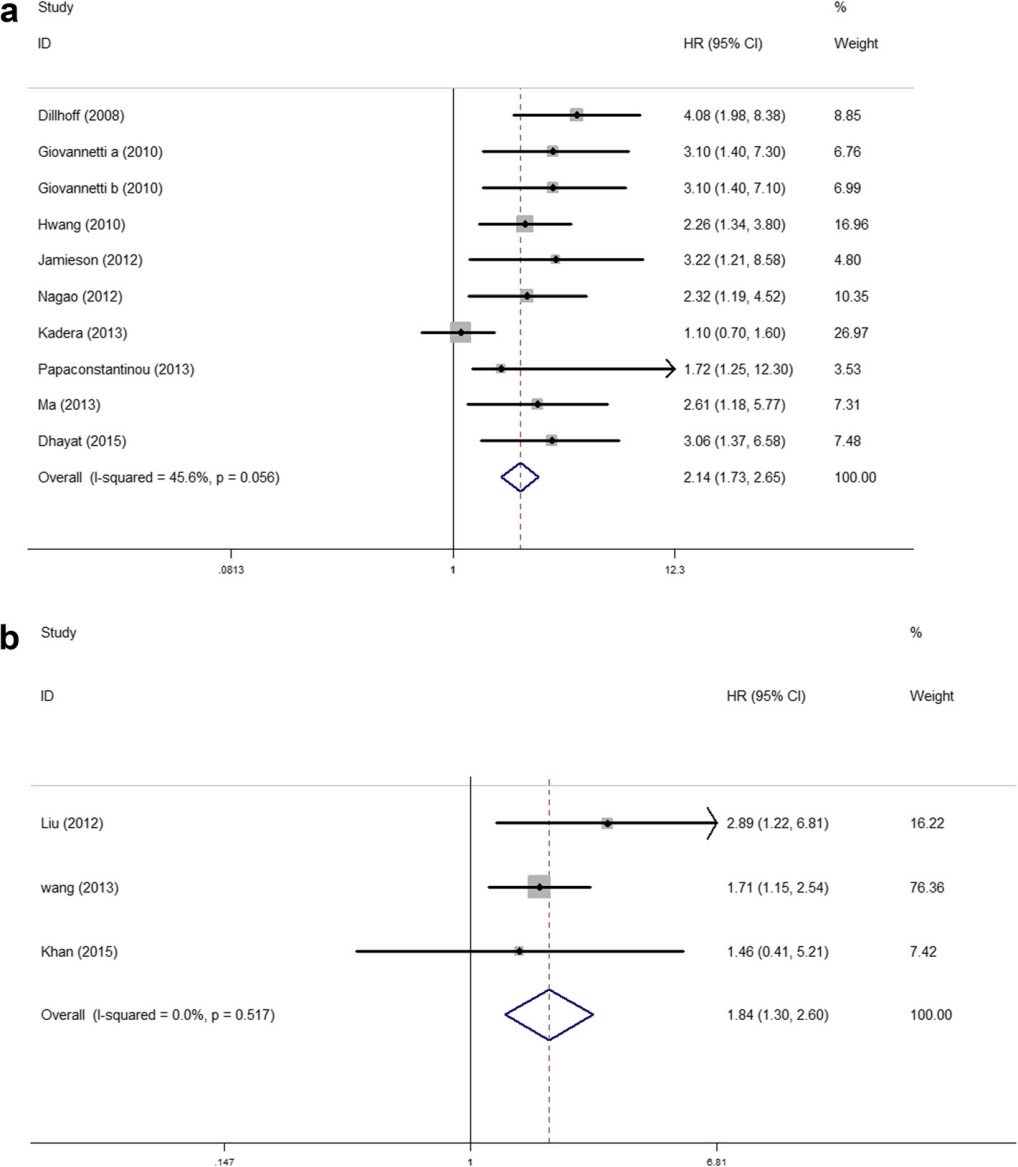
Subgroup analyses of relationship between elevated miR-21 level and OS by sample type. **a** Tissue. **b** Serum

**Fig. 5:**
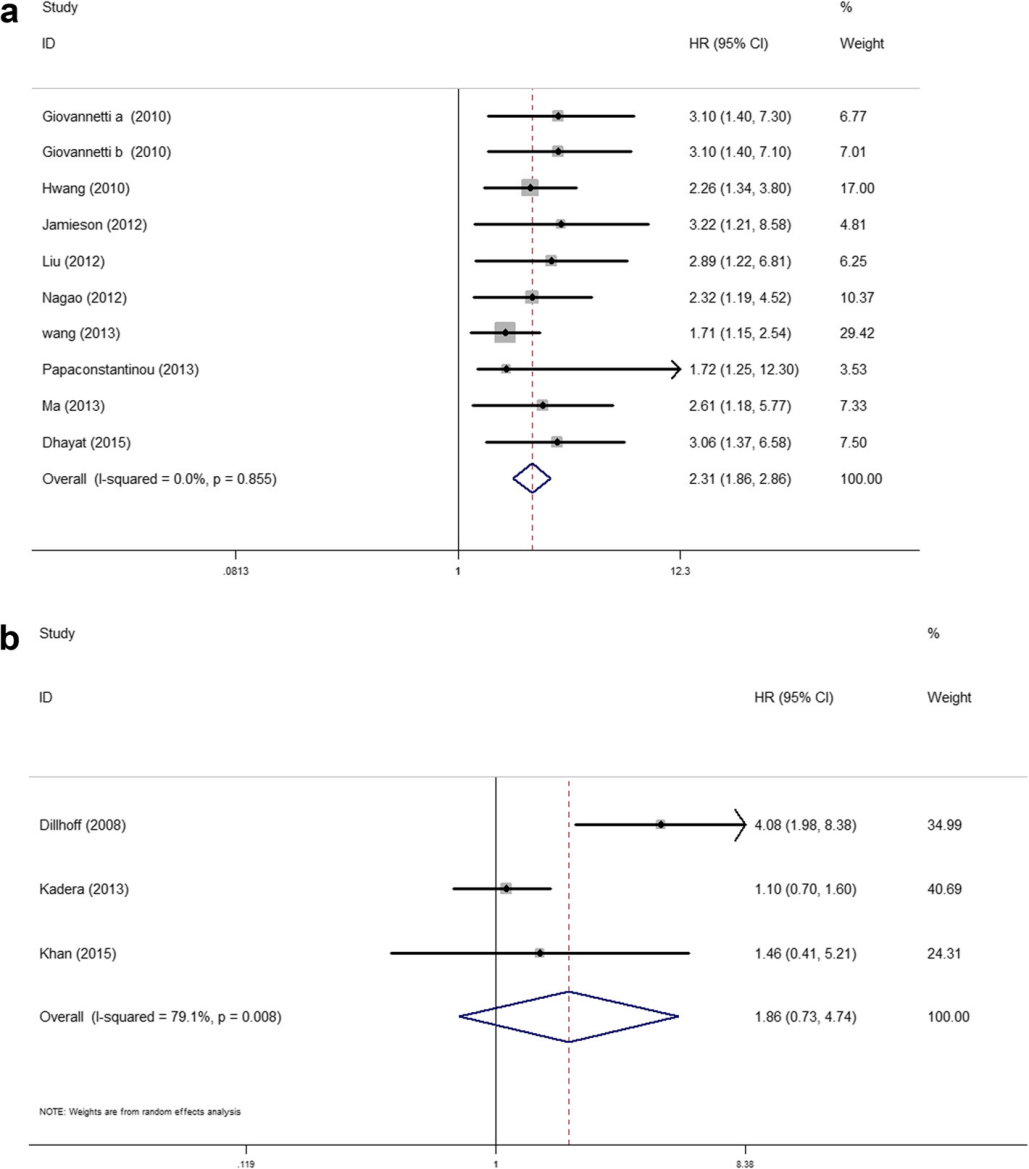
Subgroup analyses of relationship between elevated miR-21 level and OS by detection method. **a** qRT-PCR. **b** ISH

**Fig. 6:**
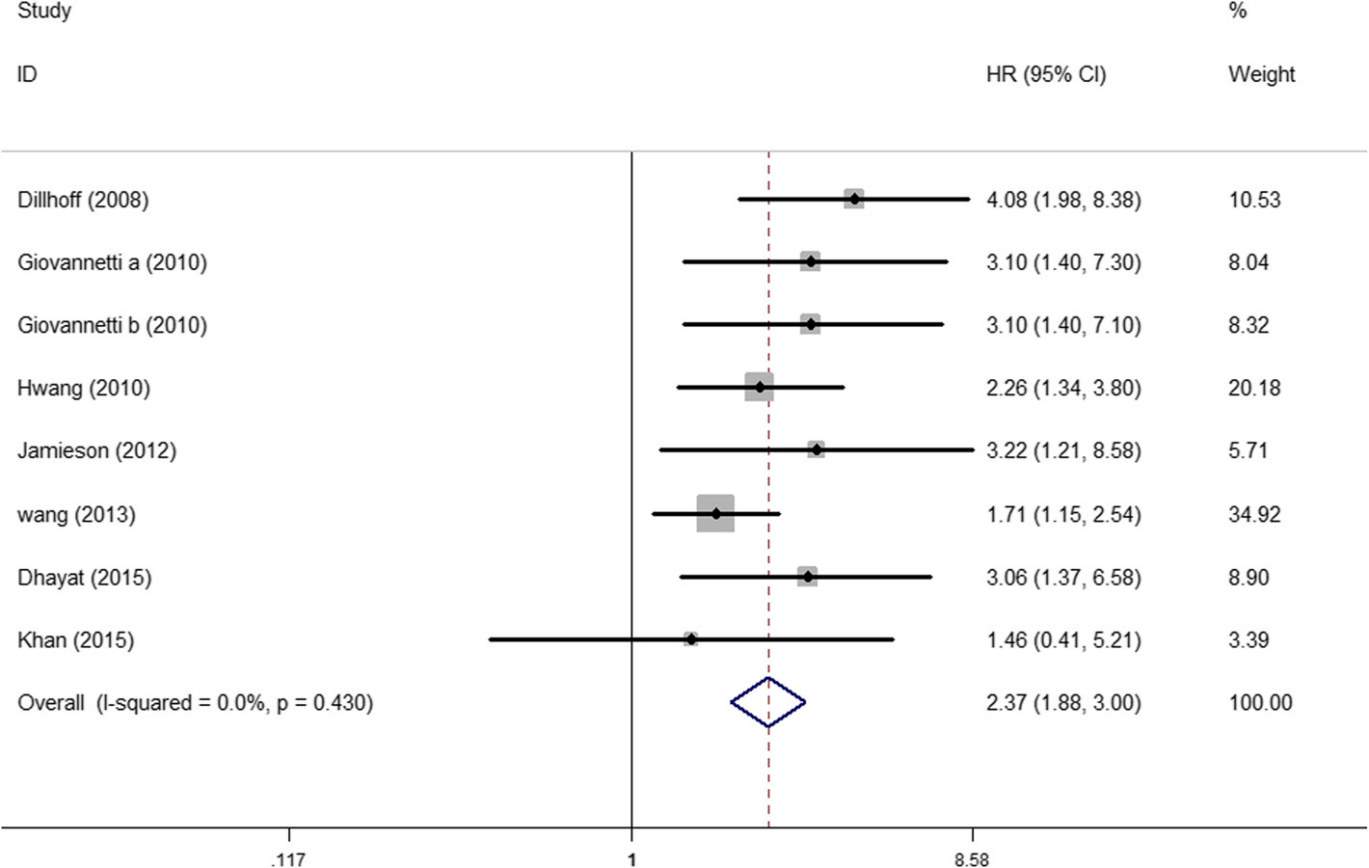
The relationship between elevated miR-21 level and OS in patients with chemotherapy

Elevated miR-21 expression was significantly predictive of poor OS (HR = 2.05, 95 % CI 1.71–2.46, *P* < 0.001), Fig. 2. In the subgroup analyses by ethnicity, no matter the cases were Asian or Caucasian, the high-level miR-21 expression was still a significantly poor predictor for OS (Asian, HR = 2.09, 95 % CI 1.62–2.71, *P* < 0.001; Caucasian, HR = 2.36, 95 % CI 1.53–3.65, *P* < 0.001), Fig. 3. We also observed that the high-level miR-21 expression was associated with poor OS both in tissue sample (HR = 2.14, 95 % CI 1.73–2.65, *P* < 0.001) and serum sample (HR = 1.84, 95 % CI 1.30–2.60, *P* = 0.001), Fig. 4. Further analyses of studies revealed that the high-level miR-21 expression detected by the qRT-PCR method predicted poor OS (HR = 2.31, 95 % CI 1.86–2.86, *P* < 0.001), while similar result did not arise in high-level miR-21 expression detected by the ISH method (HR = 1.86, 95 % CI 0.73–4.74, *P* = 0.191), Fig. 5. In patients receiving adjuvant chemotherapy, high expression of miR-21 is also associated with poor prognosis (HR = 2.37, 95 % CI 1.88–3.00, *P* < 0.001), Fig. 6.

### Correlation between miR-21 expression and clinical characteristics

There were 4 studies that reported correlations between miR-21 expression and some clinical characteristics (sex, lymph node metastasis, vascular invasion, and neural infiltration). The association between miR-21 expression level and lymph node metastasis was statistically significant (OR = 1.45, 95 % CI 1.02–2.06, *P* = 0.038). However, no significant relationship between miR-21 expression level and sex or vascular invasion or neural infiltration was observed, Table 3.

**Table 3:** Correlation between miR-21 expression and clinical characteristics

Variables	Number of studies	Model	OR(95 % CI), *P* value	Heterogeneity (I^2^, *P*-value)
Gender (male vs. female)	4	Fixed	1.05(0.75–1.47), 0.78	0.0 %, 1.00
Lymph node metastasis (positive vs. negative)	4	Fixed	1.45(1.02–2.06),0.04	0.0 %, 0.70
Vascular infiltration (yes vs. no)	3	Fixed	0.91(0.47–1.76),0.78	0.0 %,0.79
Neural infiltration (yes vs. no)	3	Fixed	1.30(0.80–2.10),0.29	0.0 %,0.67

### Publication bias and sensitivity analysis

**Fig. 7:**
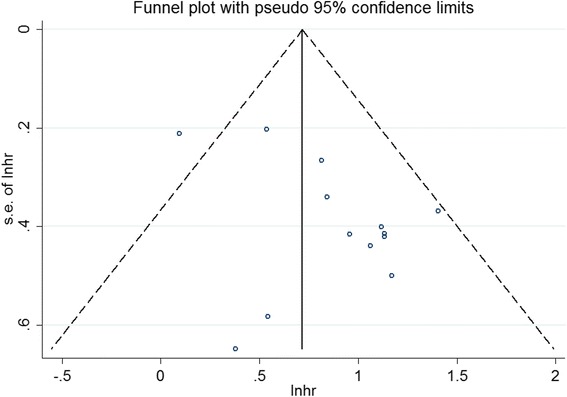
Funnel plot for publication bias analysis based on OS

The publication bias of the pooled studies was evaluated by funnel plots. Visual inspection of the funnel plots was almost symmetric in studies reported OS, Fig. 7. Sensitivity analysis was performed by exclusion of the highest weighted study and there was no individual study dominantly influenced overall HR.

## Discussion

Recently, miR-21 has gain wide attentions in cancer research for its crucial role in gene regulation and cancer development. The potential role of miR-21 as a novel diagnostic or prognostic biomarker has proved by accumulated evidence in various types of cancer, such as colon cancer, gastric cancer, breast cancer, and lung cancer [29–32]. Regarding PDAC, although the potential diagnostic effect of miR-21 had already been reported [22, 33, 34], the prognostic role is still contingent. In addition, the relation between the expression of miR-21 and patients’ clinical characteristics still needs to be defined.

In this meta-analysis, the pooled HR from 963 patients in 13 studies for OS was 2.05 (95 % CI 1.71 to 2.46, *P* < 0.01), which was considered strongly predictive [35]. Our present meta-analysis found strongly predictive value of miR-21 both in Asian and Caucasian patients; thus, we assume that it may be applied as a general prognostic marker regardless of ethnicity. An ideal prognostic biomarker should be convenient, sensitive, and credible. The majority of pooled studies detected the miR-21 expression in the tissue with qRT-PCR method. Elevated expression of miR-21 in the serum sample also predicted poor prognosis as tissue sample, which may be favored in the clinical management of PDAC. Conversely, in the subgroup analysis of ISH method, elevated expression of miR-21 did not imply poor outcomes. This result should be elaborated causing limited pooled studies using ISH method with a different cutoff value. Therefore, further studies with consistent normalization are warranted. Though we did not report the pooled relationship of miR-21 with disease-free survival, relapse-free survival, and progression-free survival due to limited studies for analysis, several studies demonstrated the significant prediction of miR-21 on these prognostic outcomes [18, 19, 27, 28]. Moreover, the patients’ clinical characteristics, including male gender, positive vascular infiltration, and positive neural infiltration showed no correlation with high miR-21 expression, but positive lymph node metastasis showed significant correlations. This important feature was consistent with several other malignancies [36, 37]. Kadera et al. reported that pancreatic cancer cells induce the fibroblasts with high expression of miR-21 and increase the invasive potential as manifested by lymph node metastasis [21].

As potential mechanisms of enhanced PDAC invasion, miR-21 has been identified to target on the phosphatase and tensin homolog (PTEN). The PTEN gene in humans generally acts as a tumor suppressor gene through the actions of its phosphatase protein product [38], which plays a critical role in cell cycle arrest, cell migration, cell spreading, and cell invasions. Aberrant expression of PTEN was associated with the development and progression of multiple tumors [39, 40]. Meng et al. found that inhibition of miR-21 in cultured HCC cells increased the expression of PTEN [41]. Giovannetti et al. reported that transfection with pre-miR-21 resulted in the reduction of PTEN expression [18], suggesting that PTEN was a direct target of miR-21. The reversion-inducing-cysteine-rich protein with Kazal motifs (RECK) is another potential target gene of miR-21 identified recently. RECK functions as a metastasis suppressor by membrane-bound inhibiting MMPs, which play an important role in extracellular matrix remodeling during tumor progression [42, 43]. Zhao et al. reported that miR-21 modulated the RECK expression through directly binding to RECK 3′-UTR [44]. Down-regulation of RECK expression was observed in tumors including PDAC when the expression of miR-21 is elevated [45, 46].

Several clinical trials have approved that adjuvant chemotherapy can effectively improve the outcome of PDAC [47, 48]. The gemcitabine or 5-fluorouracil-based chemotherapy was considered as the most effective regimen till now, while the response to the adjuvant chemotherapy remains unsatisfactory. The dense fibrotic bulk of PDAC appears to impede the delivery of chemotherapeutic drugs to cancer cells. More importantly, miR-21 expression is likely to be correlated with chemoresistance as growing evidence suggests that aberrant miR-21 expression strongly cripples the response to the chemotherapy [49–51]. The present meta-analysis revealed that elevated expression of miR-21 was associated with shorter OS in patients with chemotherapy as well. Higher expression of miR-21 was detected in PDAC cells with the higher 50 % inhibitory concentration values of gemcitabine or 5-fluorouracil [18, 19]. Meanwhile, antisense inhibition of miR-21 induced the reduction of cancer cell proliferation, invasion, and chemoresistance against gemcitabine and 5-fluorouracil [52].

Apoptotic evasion is considered to be one of the main causes of chemoresistance. Previous studies have revealed that overexpression of miR-21 can down-regulate PTEN expression and consequently activate the PI3K/Akt signaling pathway, while anti-miR-21 strategy strongly reduced phospho-AKT levels and enhanced apoptosis when used in combination with gemcitabine [18]. Furthermore, Toste et al. identified an inverse correlation between miR-21 and p85α [53], a major negative regulatory subunit of PI3K/Akt signaling [54], which was likely a potential mechanism for the known relationship between miR-21 expression and PI3K/Akt signaling. Beyond this, Wang et al. reported that miR-21 directly regulates the FasL/Fas pathway by binding the 3′-UTR region of the FasL messenger RNA (mRNA). Additionally, ectopic expression of FasL significantly abrogated the miR-21-induced chemoresistance [26]. These evidences may also partially explain that high expression of miR-21 predicts poor prognosis.

Although miR-21 holds a great promise as a novel prognostic biomarker for PADC, several limitations need to be addressed in practical application. Firstly, statistical heterogeneity among pooled studies, which may be derived from different ethnicities (Caucasian vs. Asian), sample types (tissue vs. serum), treatment (without chemotherapy vs. with gemcitabine or 5-fluorouracil), different detection methods (qRT-PCR vs. ISH), decreased the plausibility of the results. Secondly, only studies published in English were pooled in this meta-analysis which may omit some valuable articles on this issue. Thirdly, miR-21 as a novel prognostic marker of PDAC just looms in recent years, and still limited research work was done. So, the study size obtained in this meta-analysis was relatively small.

## Conclusions

In conclusion, the current meta-analysis demonstrated that miR-21 might serve as a potential prognostic marker of PDAC. Due to some limitations of this study, more complementary researches are awaited to validate our results.
